# The biogeography of kin discrimination across microbial neighbourhoods

**DOI:** 10.1111/mec.13803

**Published:** 2016-09-23

**Authors:** Susanne A. Kraemer, Sébastien Wielgoss, Francesca Fiegna, Gregory J. Velicer

**Affiliations:** ^1^ Institute of Evolutionary BiologyUniversity of EdinburghKing's BuildingsEdinburghEH9 3FLUK; ^2^ Institute of Integrative BiologyETH ZürichUniversitätstrasse 168092ZürichSwitzerland

**Keywords:** myxobacteria, social micro‐organisms, social neighbourhoods, social population structure, sociogeography

## Abstract

The spatial distribution of potential interactants is critical to social evolution in all cooperative organisms. Yet the biogeography of microbial kin discrimination at the scales most relevant to social interactions is poorly understood. Here we resolve the microbiogeography of social identity and genetic relatedness in local populations of the model cooperative bacterium *Myxococcus xanthus* at small spatial scales, across which the potential for dispersal is high. Using two criteria of relatedness—colony‐merger compatibility during cooperative motility and DNA‐sequence similarity at highly polymorphic loci—we find that relatedness decreases greatly with spatial distance even across the smallest scale transition. Both social relatedness and genetic relatedness are maximal within individual fruiting bodies at the micrometre scale but are much lower already across adjacent fruiting bodies at the millimetre scale. Genetic relatedness was found to be yet lower among centimetre‐scale samples, whereas social allotype relatedness decreased further only at the metre scale, at and beyond which the probability of social or genetic identity among randomly sampled isolates is effectively zero. Thus, in *M. xanthus*, high‐relatedness patches form a rich mosaic of diverse social allotypes across fruiting body neighbourhoods at the millimetre scale and beyond. Individuals that migrate even short distances across adjacent groups will frequently encounter allotypic conspecifics and territorial kin discrimination may profoundly influence the spatial dynamics of local migration. Finally, we also found that the phylogenetic scope of intraspecific biogeographic analysis can affect the detection of spatial structure, as some patterns evident in clade‐specific analysis were masked by simultaneous analysis of all strains.

## Introduction

Realistic models of social evolution must account for the spatial distribution of genetic relatedness and social phenotypes among potentially interacting organisms within local populations, as well as the fitness effects of their interactions (e.g. Fortunato *et al*. 2003; Vos & Velicer 2009; Wollenberg & Ruby 2009; Hawlena *et al*. 2010, 2012; Kraemer & Velicer 2014). The local biogeography of social interaction phenotypes is important because variable social environments across which individuals might readily disperse (whether passively or actively) are often powerful mediators of selection (Grafen 1984; Wolf 2003; Queller 2011). The degree to which competing social types spatially intersperse can profoundly influence the trajectory of social evolution (Kerr *et al*. 2002; Kümmerli *et al*. 2009), for example whether or not there is selection for behaviours that are beneficial only when distinct social types compete while interacting physically within mixed groups (Fortunato *et al*. 2003; Hawlena *et al*. 2010, 2012; Manhes & Velicer 2011). Similarly, at the community level, the fine‐scale spatial distribution of microbes has major implications for virulence in polymicrobial infections (Stacy *et al*. 2016). While local spatial patterns of social and genetic variation in animal populations have received considerable attention (e.g. Segurel *et al*. 2008; Hedtke *et al*. 2013) and many studies of microbial biogeography across large spatial scales have been performed (e.g. Whitaker *et al*. 2003; Nemergut *et al*. 2011), research on local spatial patterns of microbes is scarce, especially for investigations focusing on the local biogeography of sociomicrobial interactions (Fortunato *et al*. 2003; Vos & Velicer 2006; Gilbert *et al*. 2009; Stefanic & Mandic‐Mulec 2009; Wilder *et al*. 2009; Wollenberg & Ruby 2009; Sathe *et al*. 2010; Cordero & Polz 2014; Stefanic *et al*. 2015).

Kin discrimination traits may be favoured by kin selection (Hamilton 1964a,b; Sachs *et al*. 2004; West *et al*. 2006) or may originate as indirect by‐products of evolution at traits not under kin selection (Crozier 1986; Grosberg 1988; Pfennig *et al*. 1999; Smith *et al*. 2014; Rendueles *et al*. 2015b). Both of these hypotheses predict that degrees of kin discrimination between interactants should correlate positively with relevant measures of genetic distance, and several studies of social microbes have obtained results consistent with this expectation (Ostrowski *et al*. 2008; Vos & Velicer 2009; Rendueles *et al*. 2015b; Stefanic *et al*. 2015). Regardless of why kin discrimination traits originated, knowing the spatial distributions of distinct social allotypes is necessary for fully understanding the evolutionary processes of microbial social divergence in natural habitats. Both the density of social allotypes in a defined area and their spatial distribution among individuals will affect the frequency and character of inter‐ vs. within‐allotype interactions and thus the relative degree to which interactions of each category affect overall fitness.

Analysing a natural population of the fruiting social amoeba *Dictyostelium discoideum*, Fortunato *et al*. (2003) showed that distinct microsatellite genotypes often co‐occur at the millimetre scale but did not find evidence of divergence in social identity, whereas Gilbert *et al*. (2009) showed that some clonal haplotype patches of *D. discoideum* can reach several metres in size. Gilbert *et al*. (2007) showed that most *D. discoideum* fruiting bodies are clonal for microsatellite haplotypes, and Ostrowski *et al*. (2008) found that the developmental compatibility of natural *D. discoideum* isolates decreases with genetic distance between strains (again based on microsatellite genotyping), but these findings were not examined in a biogeographic context. Vos & Velicer (2008a) showed that natural populations of the social bacterium *Myxococcus xanthus* are structured across large spatial scales at conserved multilocus sequence tags (MLST). Moreover, the same authors (Vos & Velicer 2009) demonstrated that pervasive antagonisms during multicellular fruiting body development are more pronounced among more divergent isolates from globally disparate origins than among more closely related isolates from the same cm‐scale population. However, those studies did not examine either genetic diversity or the character of interactions across a range of small scales within which migration and interaction are expected to be high. Working with the Gram‐positive bacterium *Bacillus subtilis,* Stefanic & Mandic‐Mulec (2009) documented the coexistence of four quorum‐sensing pherotypes within 1‐cm^3^ soil samples, and, more recently, Stefanic *et al*. (2015) found twelve colony‐merger kin discrimination allotypes among the same isolates, but did not analyse the distribution of those allotypes across multiple spatial scales.

*Myxococcus xanthus* is a broadly distributed soil bacterium (Vos & Velicer 2008a) that exhibits genetically and behaviourally complex social traits throughout its life cycle (Dworkin & Kaiser 1993), including social gliding motility (Youderian *et al*. 2003; Youderian & Hartzell 2006; Sun *et al*. 2011), cooperative predation (Berleman *et al*. 2008) and, upon starvation, the social development of multicellular fruiting bodies that harbour metabolically quiescent spores (Kuner & Kaiser 1982; Zusman *et al*. 2007). All of these social traits are highly variable among natural isolates (Krug *et al*. 2008; Vos & Velicer 2008b; Kraemer *et al*. 2010). In one local population, 78 cm‐scale isolates were found to frequently be antagonistic towards one another (Vos & Velicer 2009; Rendueles *et al*. 2015a,b) and to include at least 45 kin discriminatory allotypes (Vos & Velicer 2009). However, the frequency of social compatibility and degrees of genetic relatedness among cells within vs. across neighbouring fruiting bodies and fruiting body neighbourhoods remain unknown.

Here we characterize the spatial distribution of both kin discriminatory allotypes and genetic relatedness between interactants across fine‐scale microbial neighbourhoods within which the potential for both active and passive dispersal is great (Vos & Velicer 2008a), as well as across larger scales. Our spatial scales begin at the low end with isolates taken from within discrete groups of actively cooperating *Myxococcus* cells, namely fruiting bodies, at ~1–10 cell‐body lengths (~10–100 μm) and extend to neighbouring fruiting bodies separated by millimetres, then to neighbourhoods of fruiting bodies separated by centimetres and finally to soil patches separated by metres and kilometres.

We use colony‐merger incompatibility as our criterion for defining social allotypes. In several species of motile bacteria, colony‐merger compatibility can be determined by whether adjacent swarming colonies on an agar plate exhibit a reduced propensity to freely merge relative to colonies of the same genotype (Gibbs *et al*. 2008; Vos & Velicer 2009; Rendueles *et al*. 2015b; Stefanic *et al*. 2015). In *M. xanthus,* such colony‐merger incompatibilities have been shown to be associated with a reduced propensity of cells from adjacent colonies to co‐aggregate into shared fruiting bodies (Rendueles *et al*. 2015b) and thus are likely to play a major role in structuring the local diversity of natural soil populations. Using this criterion, we estimate the probability of meeting socially or genetically identical neighbours as a function of hypothetical migration distance. The joint analysis of both genetic relatedness and social compatibility across several metric scales within local populations allows us to map the social structure of microbial neighbourhoods.

## Materials and methods

### Sample collection

**Figure 1:**
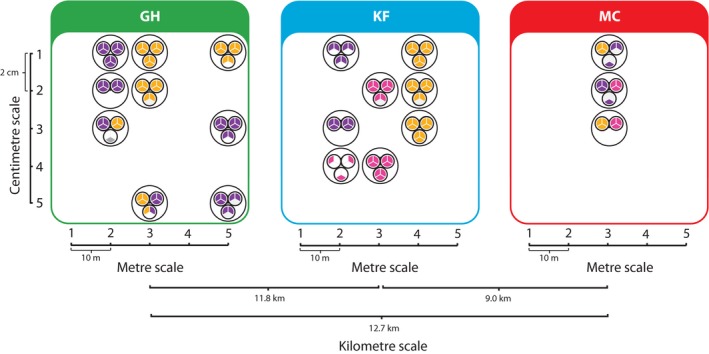
Phylogenetic clade members are nonrandomly clustered at multiple spatial scales. Biogeographic structure becomes evident when clade membership from a phylogenetic tree (Fig. S1, Supporting information) is mapped onto the hierarchical sampling layout. Samples are derived from three major sites separated pairwisely by ~9–13 km (GH, KF and MC). Large circles within each site represent soil cores on which fruiting bodies (small circles) formed. Neighbouring fruiting bodies from the same soil core were separated by millimetres. Individual clones (sectors within the small circles) were isolated from each fruiting body and genotyped for five loci (*N *=* *147; Table S1, Supporting information). Clone sector colours indicate the phylogenetic clade to which each clone belongs (Fig. S1, Supporting information; Clade 1—orange; Clade 2—purple; Clade 3—pink; outlier clone—grey).

*Myxococcus xanthus* clones were isolated from soil samples in a nested design comprising micrometre, millimetre, centimetre, metre and kilometre scales (Kraemer & Velicer 2011). More specifically, we sampled within each of three undeveloped forested areas located near Bloomington, Indiana, USA, that are separated pairwisely by distances of ~9.0–13 km (Moore's Creek (MC) and Kent Farm (KF) sites of the Indiana University Research and Teaching Preserve (http://www.indiana.edu/~preserve/preserve.shtml) and forested, undeveloped private property (GH), GPS coordinates available upon request). Within each of these three km‐scale sites, we collected 25 soil cores within five clusters of five cores each, with clusters spaced at ten‐metre intervals along a line and cores within each cluster spaced at two‐centimetre intervals (Fig. 1). For this study, we originally sought to analyse three of the linear transects separated by tens of metres (m scale) within each km‐scale site (GH, KF and MC), three soil cores separated by centimetres (cm scale) along each m‐scale transect, three fruiting bodies separated by millimetres (mm scale) from each soil core and three independent isolates from within each fruiting body (μm scale). This design would have ideally yielded a total of 243 (3^5^) independent isolates for analysis. However, a lack of fruiting bodies in some transects or soil cores and sequencing difficulties with some isolates limited our final analysed sample set to 147 fully genotyped isolates from a spatial distribution represented in Fig. 1 (see also Results).

Isolate names identify their spatial origin. For example, clone KF3.2.8A represents one of three isolated clones (A–C) sampled at the Kent Farm location, at m‐scale site 3, cm‐scale soil core 2, from fruiting body number 8 (with fruiting bodies separated by millimetres within a soil core). For centimetre, metre and kilometre scales, we calculated average distances from known intersample distances. For micro‐ and millimetre scales, precise measurements were not feasible, so average distances among single cells were estimated indirectly by assuming that each clone was on average maximally separated from another by a third of the respective diameter for both fruiting bodies (~100 μm average diameter assumed) and soil cores (~0.9 cm diameters). Hence, we infer the following average distances among isolates at each scale: micrometres (0.000033 m); millimetres (0.003 m); centimetres (0.036 m); metres (18 m); kilometres (11 000 m). We use these values for all of our subsequent analyses and in all figures, but refer to them only by their respective categorical names.

### Genotyping: multilocus sequence tags (MLST)

Primer pairs were previously designed to amplify fragments of the loci *Mxan_0128*,* Mxan_0533*,* Mxan_0396*,* Mxan_4405* and *Mxan_5783* (*i.e. pilA*) of the laboratory strain DK1622 (Kraemer & Velicer 2011). However, we found that the primers for the DK1622 allele of *Mxan_0396* exclusively amplified a similar‐sized fragment of a different locus, *Mxan_1277*, in all natural isolates sampled in Indiana, USA, including those isolates analysed previously by Kraemer & Velicer (2011). Thus, sequences reported as *Mxan_0396* (Kraemer & Velicer 2011) actually represent *Mxan_1277*. DNA isolation and sequencing was performed as previously described (Kraemer & Velicer 2011). Genotyping was successful for a total of 147 clones derived from 57 fruiting bodies within 20 soil cores (Table S1, Supporting information) at five highly variable chromosomal loci (*Mxan_0128*,* Mxan_0533*,* Mxan_1277*,* Mxan_4405* and the *pilA* motility gene *Mxan_5783*). Clones for which we failed to obtain sequences for all loci were excluded from the analyses. Sequences for each locus were aligned individually based on their translated amino acid sequences in mega version 5.1 (Tamura *et al*. 2007) and finally concatenated to a single sequence comprising a total of 1445 bp, including gaps. Sequence type numbers and nucleotide diversity for each nested sample were estimated in dnasp version 5 (Librado & Rozas 2009). DNA sequences are available on GenBank (Accession nos: KX690652‐KX691394). We tested the relative strengths of recombination and selection in the data set, as well as the relative contributions of recombination and mutations to the variation observed.

### Phylogenetic analyses and population structure

We chose the most likely model of sequence evolution for the concatenated sequence, HKY85 + I, based on the Akaike information criterion in modelgenerator version 0.81 (Keane *et al*. 2006) and subsequently inferred phylogenetic relationships among both the 26 unique sequence types (STs) and the 147 individual clones based on their concatenated sequences using maximum‐likelihood estimation in phyml version 3.0 (Guindon *et al*. 2010) with 1000 and 100 bootstrap replicates, respectively. We assessed the potential influence of recombination and selection in our data set comprised of the 26 unique sequence types (Table S1, Supporting information).

We tested for population structure by correlating pairwise genetic and geographic distances with one another. Pairwise genetic distances between isolates were calculated using the maximum composite likelihood of base substitution (MCL) in mega version 5.1 (Tamura *et al*. 2007). As pairwise differences are not always independent from one another, we tested for significant deviations from the null model of no correlation among pairwise distances by applying a Mantel test (Mantel 1967) with 9999 permutations (two‐sided *t*‐test) as integrated in the r package *ade* *4* (Dray & Dufour 2007). Moreover, post hoc tests of genetic differences also suffer from the same potential problem of data interdependence. To circumvent this problem, we developed a customized quasi‐random sampling approach based on draws of three times four isolate pairs per spatial scale without replacement in r version 3.1.0 (R Core Team 2014) that prevented any individual isolate from being represented in more than one strain pair among all analysed pairs. Thus, all sampled pairs both within and across scale comparisons are fully independent of one another for all replicates. From these independent replicate sample sets, we estimated the probabilities of paired isolates being genetically identical as a function of geographic distance between sample points. The code for this method is available upon request. Subsequently, we performed one‐way ANOVA and a post hoc Tukey's HSD to test for significant differentiation across independently sampled metric scales.

### Recombination and selection

To test for recombination, we applied Genetic Algorithm Recombination Detection (GARD) using the appropriate model of sequence evolution (Pond *et al*. 2006). After establishing that four of five sequences showed signs for recombination, we applied the Kishino–Hasegawa (KH) test (Hasegawa & Kishino 1989) for assessing whether or not the phylogenies inferred from sequences left of the recombinational break points agree with those inferred from the sequences to the right. Due to the presence of recombination in the data set, tests for selection were performed utilizing the PARRIS method (Scheffler *et al*. 2006). All tests are implemented in the package hyphy (Pond *et al*. 2005) on the Datamonkey server (Pond & Frost 2005; Delport *et al*. 2010;). Finally, we assessed the probable impact of the detected recombination on our phylogenetic inference in three ways. First, we applied the ClonalFrame model to infer the consensus tree (Didelot & Falush 2007). Second, we removed the *Mxan_0128* locus from the concatenated sequence and re‐inferred the phylogeny, as this was the only gene that showed highly significant disagreement of phylogenies flanking the recombination break points according to the KH test (Table S2, Supporting information). Third, we inferred a phylogenetic network using SplitsTree4 (Huson & Bryant 2006) to test whether recombination leads to a more network‐like topology (Fig. S3, Supporting information).

### Estimation of recombination and mutation rates

We used the software ClonalFrame (Didelot & Falush 2007) to estimate the relative contributions of recombination and mutation to genome evolution based on the unique sequence types to avoid skewed sampling of the tree due to the presence of identical clones (Vos & Didelot 2009). In particular, we estimated the ratios of recombination vs. mutation rates, as rho/theta and r/m, respectively. Independent runs were compared with one another to ensure parameter convergence highlighted by a Gelman–Rubin statistic of <1.1 (Vos & Didelot 2009). All final results are based on 500 000 MCMC iterations after a burn‐in period of equal length. Parameter values have been recorded at 100 iteration step intervals (parameters for the run: −*x* 500 000; −*y* 500 000, −*z* 100).

### Kin discrimination assays

For each of the 60 independently sampled isolate pairs used to examine the relationship between genetic relatedness and interorigin distance, we tested for the presence of kin discrimination phenotypes at the interface of oncoming colony swarms (Vos & Velicer 2009). All strains were inoculated on CTT 1.5% agar plates and incubated at 32 °C, 90% rH for 4–5 days. An inoculum from each strain was transferred into eight ml of CTT liquid and grown for approximately 24 h at 32 °C, 300 rpm in an orbital shaker. The kin discrimination assay was performed on low‐Casitone CTT 1.5% agar plates (0.1% and 0.3% Casitone). Liquid cultures were harvested at mid‐exponential phase by centrifugation (4500 ***g***, 15 min) and resuspended in TPM buffer to ~5 × 10^9^ cells/mL. We initiated each assay by inoculating four 10‐μL spots on a plate at 10‐mm distances in a square configuration, with each spot adjacent to the other spot of the same isolate and one spot of the other isolate. After incubation for 6 days at 32 °C, 90% rH, colony‐encounter interfaces between distinct isolates were compared to those between colonies of the same isolate (Rendueles *et al*. 2015b). Colony‐interface phenotypes that were visibly altered by interaction between distinct isolates relative to self–self interfaces were scored as kin discrimination phenotypes. Such phenotypes took the form of colony‐territory boundaries that were absent from self–self interfaces and/or altered patterns of fruiting body formation along non‐self interfaces relative to self–self interfaces. Each of the three replicate sample sets included four isolate pairs per spatial‐scale category and five control plates in which all four spots were of the same isolate (with each plate having a different isolate). To avoid scoring biases, swarm‐encounter assays were carried out independently by three investigators (S.W., F.F. and G.J.V.) who were blind to strain‐pair identities during scoring. Interface phenotype classifications were unanimous for 147/150 pairwise assays. In the three cases of score disagreement, the majority classification was adopted.

## Results

### Sample isolation, genotyping results and phylogenetic inference

For this study, 176 clonal isolates of *M. xanthus* were originally sampled from a total of 59 individual fruiting bodies across 20 soil cores, but only 147 of these isolates were successfully genotyped for five genes previously found to be highly variable among natural isolates (Kraemer & Velicer 2011). The successfully genotyped isolates were derived from 57 individual fruiting bodies. Three fruiting bodies were sampled from most soil cores that yielded any at all, and three clones were isolated from most fruiting bodies (Fig. 1, Table 1). Isolates were successfully obtained from either two or three cm‐scale soil cores from each of three m‐scale transects at the KF and GH locations, but only one m‐scale transect at the MC location yielded fruiting bodies. Among the 147 five‐gene sequence concatemers, we identified 26 unique sequence types (STs, Table S1, Supporting information). For purposes of this study, we consider isolates that share an ST to be genetically identical, although it is very likely that such isolates vary at other loci. STs clustered into three distinct clades of a phylogenetic tree (Figs S1 and S2, Supporting information). When clade membership is mapped across our hierarchical sampling design, it is evident that clade members are not randomly interspersed, but rather are clustered at multiple levels (Fig. 1).

**Table 1:** Sample sizes, sequence types and nucleotide diversities across three hierarchically probed sampling locations

Sample sets	*N*	Sequence types (STs)	Nucleotide diversity (π)
*Kilometre scale*			
Bloomington area (3 km‐scale sites)	147	26	0.050
*Metre scale*			
GH (3 m‐scale sites)	70	11	0.035
KF (3)	58	9	0.063
MC (1)	19	6	0.063
*Centimetre scale*			
GH2 (3 cm‐scale cores)	22	3	0.021
GH3 (3)	25	5	0.021
GH5 (3)	23	4	0.030
KF2 (3)	15	4	0.038
KF3 (2)	17	2	0.007
KF4 (3)	26	3	0.002
MC3 (3)	19	7	0.063
*Millimetre scale*			
GH21 (three fruiting bodies)	9	1	0.000
GH22 (2)	6	1	0.000
GH23 (3)	7	3	0.045
GH31 (3)	9	3	0.003
GH32 (3)	8	2	0.003
GH35 (3)	8	3	0.039
GH51 (3)	8	2	<0.001
GH53 (3)	8	1	0.000
GH55 (3)	7	1	0.000
KF21 (3)	6	1	0.000
KF23 (2)	6	1	0.000
KF24 (3)	3	2	0.008
KF32 (3)	8	1	0.000
KF34 (3)	9	2	0.007
KF41 (3)	9	2	0.005
KF42 (3)	8	1	0.000
KF43 (3)	9	2	0.001
MC31 (3)	6	3	0.040
MC32 (3)	7	3	0.060
MC33 (2)	6	3	0.060

### On the impact of recombination and selection on phylogenetic inference

Polymorphism patterns at the variable loci sequenced here are influenced by recombination (Table S2, Supporting information), which we detected in all loci except for *Mxan_4405*. Importantly, recombination events are not likely to have interfered with our phylogenetic analyses for several reasons. First, a clonal frame analysis that allows for the presence of recombination in phylogenetic inference yielded an almost identical tree topology to that derived from a maximum‐likelihood approach. This is highlighted by adding those bootstrap values from the ClonalFrame analysis in Fig. S1 (Supporting information) that support the equivalent branches among both trees, that is branches leading to areas of the tree harbouring the same set of taxa. Second, we found no significant differences in the topologies of trees inferred from left and right of each estimated recombination break point within genes, with the exception of *Mxan_0128*. The exclusion of this gene from phylogenetic inference yielded the same tree as depicted in Fig. S1 (Supporting information, data not shown). Third, the overall divergence into the initially observed three clades was reconfirmed by phylogenetic network analysis in SplitsTree (Fig. S3, Supporting information), which suggests that our tree captures the phylogenetic relationships reasonably well. While recombination does not undermine the robustness of our phylogenetic inference, its impact on sequence evolution was nonetheless extensive. The mutation rate θ within our data set is estimated to be approximately fourfold higher than the recombination rate ρ (ρ*/*θ* *= 0.26). However, the average number of nucleotides that were changed per recombination event was estimated as ~12. Thus, overall, recombination had approximately threefold greater impact in introducing new variation per event than mutation (r/m = 3.4). Finally, no evidence of selection (while also accounting for recombination) was detected (Table S2, Supporting information).

### Within‐group relatedness is very high, but social identity begins to transition across adjacent fruiting bodies at the millimetre scale

**Figure 2:**
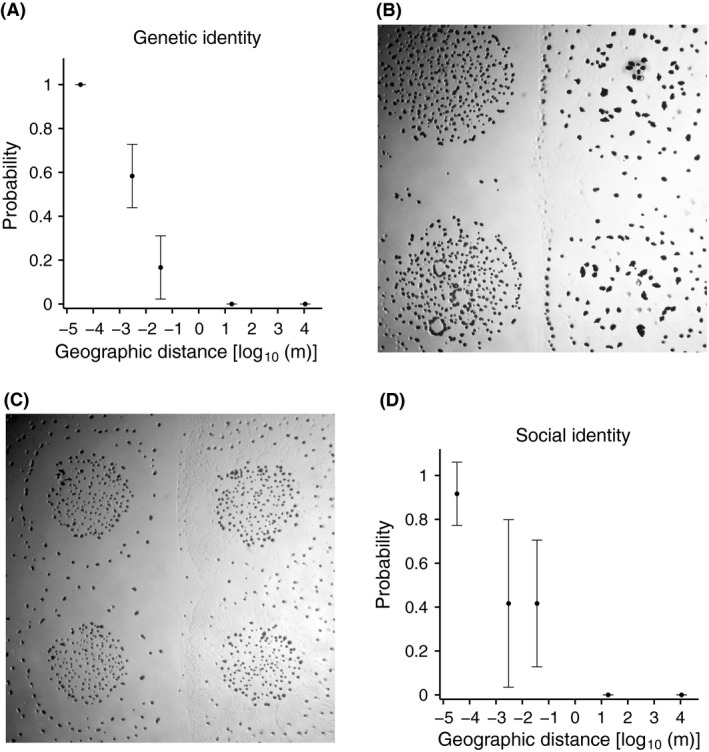
Genetic and social relatedness both decrease precipitously with increasing hypothetical migration distance. (A) The probability that any two randomly sampled isolates (*N *=* *60 pairs sampled without replacement) share the same concatemer sequence type is maximal within fruiting bodies and decreases significantly with each expansion of spatial scale up to metres (*N *=* *3 replicates comprising four pairs each per metric scale). (B) and (C) Examples of colony‐interface phenotypes of four strains that differed between encounters of self (horizontal interfaces) vs. encounters of nonself (vertical interfaces). (B) Strains GH227C (left) and KF322B (right). (C) Strains GH217A (left) and strain MC338B (right). For each of the four strains in the (B) and (C) pairs, its vertical colony‐interface (encounter of nonself) phenotype is markedly different than its horizontal interface (encounter of self) phenotype. (D) The probability that any two randomly sampled isolates (the same as used for panel *A*) belong to the same social swarming allotype also decreases at and beyond the interfruiting body scale (millimetres) and correlates strongly with the probability of genetic identity (Pearson's *r *=* *0.94; *P *=* *0.02). All error bars depict standard deviations.

Using a subset of all isolates, we analysed the relationships between spatial distance and both genetic and social relatedness. For both analyses, we quasi‐randomly sampled isolate pairs to generate three fully independent replicate pair sets at each spatial scale (*N = *4 pairs per spatial scale per replicate; *N*_TOTAL_ = 60 pairs). In the genetic analysis of this subset of 120 isolates, isolates sampled from the same fruiting body were genetically identical in all pairs. However, the proportion of genetically identical pairs decreased greatly with each stepwise increase of scale up to the metre scale, at which all pairs were composed of genetically distinct isolates (which was also the case at the kilometre scale (Fig. 2A; millimetre ~58% within‐pair identity, *P* = 0.0017 for difference from micrometre scale; centimetre ~17%, *P *=  0.0017 for difference from millimetre scale; metre 0%, *P* = 0.24 for difference from centimetre scale; all *P*‐values derived from a post hoc Tukey's HSD test among individual metric scales). Thus, average genetic relatedness among members of the same fruiting body social group tends to be very high, but the likelihood of migrants encountering social partners of the same genotype drops precipitously for even the shortest migration events across neighbouring fruiting body groups and decreases further with increasing distance, with effectively no chance of meeting individuals of the same ST after m‐ or km‐scale migration events.

To analyse patterns of social identity, we observed colony–encounter phenotypes to score whether distinct isolates belonged to the same social allotype or different allotypes (see Materials and methods). Only if the colony‐interface phenotype of either paired isolate was clearly altered by encountering the other (e.g. if colonies did not freely merge), the two isolates were classified as distinct allotypes (e.g. Fig. 2B, C). Overall, 39 of the 60 isolate pairs exhibited kin discrimination (KD) phenotypes, but these were found predominantly among isolate pairs from the larger sampling scales. Average social relatedness, that is the probability of social identity between randomly sampled isolates, correlated negatively with inter‐sample‐origin distance (Fig. 2D, Pearson's *r* = 0.94; *P *= 0.02). More specifically, isolates sampled from the same fruiting body almost always belonged to the same discrimination group (1/12 μm‐scale pairs showed a KD phenotype). Social relatedness among the mm‐ and cm‐scale pairs was similar and intermediate (7/12 KD pairs each) but decreased to zero at the metre and kilometre scales (0/12 KD pairs each).

### The size range of genetically homogenous patches

When considering all isolates within the total sample set at each spatial scale, genetic homogeneity was the norm for most fruiting bodies and often extended to millimetre‐scale patches but never to centimetre‐scale patches (Table S1, Supporting information). Most fruiting bodies (micrometre scale) represented by more than one isolate were genetically homogeneous (42/51, 82%). The remainder tended to harbour very low levels of diversity (π = 0.0007–0.0220), with one exception, GH3.5.2, which included two isolates that differed by a large genetic distance of π = 0.076. Only a minority of mm‐scale patches containing multiple fruiting bodies was pure for a single ST (8/20, 40%) and none of the seven cm‐scale soil core sample sets were genetically homogeneous (Table 1).

Fruiting bodies with multiple isolates (*N = *2 or 3) harboured only a single ST in the vast majority of cases (42/51), and the remaining nine fruiting bodies harboured at most two STs. The mm‐scale soil‐core sets (*N = *3–9) included up to three STs, centimetre‐scale sample sets (*N = *15–26) contained from two to seven STs and the three m‐scale sets (*N *=* *19–59) contained eleven, nine and six STs (GH, KF and MC, respectively; Table S1, Supporting information). Only one ST (ST13) was present at more than one m‐scale site, and no ST was represented at more than one km‐scale site (Table S1, Supporting information).

**Figure 3:**
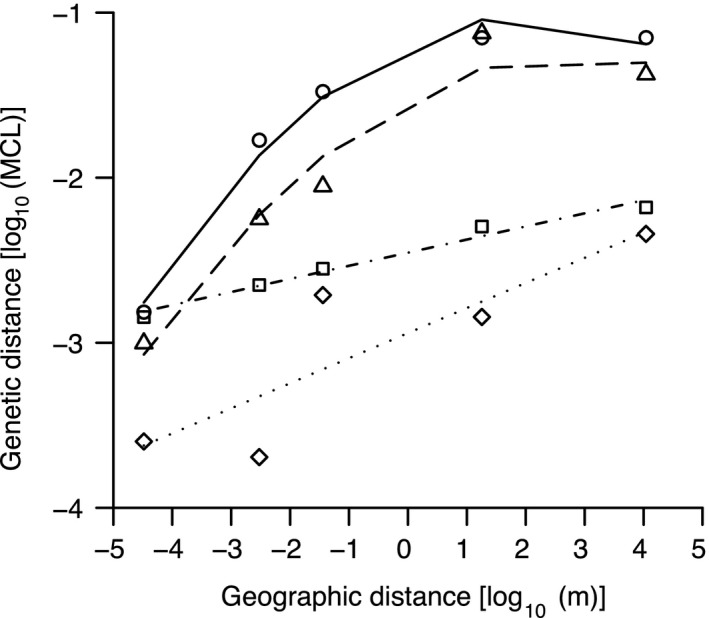
Geographic‐ vs. genetic‐distance relationships can be clade specific. Symbols represent mean pairwise genetic distance at each scale for the entire data set combined (circles) and for each of the three major phylogenetic clades. Best‐fit lines reflect second‐order polynomials for the total set and Clade 3 (triangles) and linear regression for Clades 1 and 2 (squares and diamonds, respectively). MCL = maximum composite likelihood of base substitution in mega version 5.1 (Tamura *et al*. 2007).

The negative relationship between genetic relatedness and spatial distance is also inversely reflected by a plot of average pairwise genetic distances among all possible isolate pairs at each sampling scale (Fig. 3, circles). Mean pairwise genetic distance is nearly zero within fruiting bodies, increases ten‐fold across neighbouring fruiting bodies (millimetre scale) and increases successively twofold further at both the centimetre and metre scales before reaching a plateau between the metre and kilometre scales. The positive relationship between genetic and geographic distance is extremely significant for the total sample set and all three major clades considered separately (*P *<* *0.0001 each; Mantel tests).

### Phylogenetic analysis of population structure

In a manner similar to a previous study that employed highly conserved marker loci (Vos & Velicer 2008a), a simple plot of mean pairwise genetic distance vs. spatial distance does not suggest spatial structuring beyond the metre scale among this study's isolates (Fig. 3). However, the distribution of STs and clade members across our three km‐scale sample sites reveals that genetic variation is in fact spatially structured at this broadest scale as well. First, no members of Clade 3 (the oldest clade, Figs S1–S3, Supporting information) were found at the GH site, a result extremely unlikely to occur by chance (*P < *10^−5^, binomial test, Figs 1, S1, Supporting information). Second, at the two km‐scale sites harbouring most samples (GH and KF), the frequency distribution of the largest two clades (Clades 1 and 2) deviated significantly from random expectations (binomial tests, *P *<* *0.01). Third, when STs were each paired with the phylogenetically nearest distinct ST, the frequency of those pairs in which both members originated from the same kilometre‐scale site was significantly greater than the random expectation assuming no spatial structure at the kilometre scale (exact binomial tests, *P *<* *0.001). Finally, the average genetic distance among STs in the same clade from the same km‐scale site was significantly lower than the average genetic distance among members of the same clade across all km‐scale sites (Wilcoxon rank‐sum tests of 50 (Clades 1), 30 (Clade 2) or 15 (Clade 3) quasi‐randomly resampled distance values from within or among sites, all *P *<* *0.01).

Focusing our analysis on the scale step from metres to kilometres, average genetic distance (Fig. 3) between members of Clade 1 and Clade 2 increased significantly [Clade 1: 0.0015 (m) to 0.0058 (km), *P *<* *0.001 (Wilcoxon rank‐sum test); Clade 2: 0.0013 (m) to 0.0049 (km), *P *<* *0.001 (Wilcoxon rank‐sum test)]. However, there was no increase for Clade 3 [0.0732 (m) to 0.0428 (km)]. Thus, genetic distance considered alone suggests that genetic variation increases across all sample‐scale intervals for Clades 1 and 2 but only from micrometre to metre scales for Clade 3 (Fig. 3). Because pairwise distances in Clade 3 are much higher at the metre and kilometre scales than in the other two clades in the combined data set, Clade 3 data points mask the rise in genetic diversity across the two largest scales for Clades 1 and 2 that are evident when the clades are considered separately (Fig. 3). Thus, fine gradations in the phylogenetic scope of biogeographic analyses can strongly affect what patterns are detected.

At our smallest spatial scale of analysis within fruiting bodies, we note that genetically polymorphic fruiting bodies contained distinct sequence types from the same clade in all but one case. In fruiting body GH3.5.2, clone A belongs to Clade 2, whereas clone B represents Clade 1. This exceptional case suggests that highly distinct genotypes can occasionally co‐aggregate into a common fruiting body, but this appears to occur very rarely.

Finally, the slope of the relationship between genetic distance and geographic distance differs among the three clades across smaller spatial scales, most strikingly between the steepest slope of Clade 3 and the smaller slopes of the other two clades (Fig. 3). This difference suggests that there are biological traits (e.g. territorial kin discrimination) that prevent free mixing of substantially distinct genotypes into the same fruiting body group, even when highly divergent genotypes coexist at the millimetre scale. Such traits would restrict within‐group diversity of Clade 3 fruiting bodies to levels similar to fruiting bodies from the other two clades and thus cause the steeper slope of genetic vs. geographic distance for Clade 3. Also, there are several instances in which the area of what appear to be sequence‐type patches extends to the millimetre or centimetre scale (e.g. ST2, ST3 and ST22, Table S1, Supporting information), which would steepen the slope of this relationship from those scales to the metre scale.

## Discussion

We are only beginning to understand the relative action of forces that either limit or promote the dispersal of microbes from local to global spatial scales and that determine lineage fates after dispersal events, that is the forces that shape microbial biogeography (Fierer 2008; Nemergut *et al*. 2011; Hanson *et al*. 2012). Because social and community interactions are often a major component of microbial fitness (Fortunato *et al*. 2003; Kiers & Denison 2008; Hawlena *et al*. 2012), the biogeographic distributions of microbes strongly shape their selective landscape. This includes the landscape of cooperative and noncooperative interactions among conspecifics that strongly drive social evolution. Thorough characterization of microbiogeographic patterns *per se* is a first step towards better understanding the forces that shape those patterns, including the heterogeneous habitats of soils (Nunan *et al*. 2003; Grundmann 2004).

### Pattern: a diverse mosaic of social allotype patches begins at the millimetre scale

*Myxococcus xanthus* cells have great potential to disperse locally by active motility (Vos & Velicer 2008a) and/or passive mechanisms such as water flow and carriage by animals (e.g. Figuerola & Green 2002; Dahl *et al*. 2011). However, we found natural populations to be spatially structured at all scales beyond an individual fruiting body, including the smallest examined scale that encompasses multiple adjacent social groups (millimetres). Both social relatedness and genetic relatedness are extremely high within discrete fruiting bodies at the micrometre scale but much lower already at the millimetre scale. Thus, our data indicate that even very short dispersal events of a few hundreds of cell lengths will often lead to encounters with cells of a different kin discrimination allotype. The sampled populations form a fine‐scale mosaic in which patches of distinct social types often coexist among directly adjacent fruiting bodies at the millimetre scale and in which contiguous patches of the same allotype sometimes extend to the centimetre scale but never to the metre scale. This fine scale of social patchiness implies the existence of an immense number of *M. xanthus* allotypes worldwide, which in turn strongly suggests that a large number of genetic loci contribute to defining *M. xanthus* social identity (Rendueles *et al*. 2015b). Recently, a similar inference was made from the high diversity of *B. subtilis* social allotypes found within small soil samples (Stefanic *et al*. 2015; Lyons *et al*. 2016).

Social interactions between *M. xanthus* allotypes appear to play a major role in limiting successful intergroup migration, including kin discrimination territory boundaries such as those examined here. First, as highlighted here and in other studies, colonies of distinct social allotypes exhibit a reduced propensity to merge (Vos & Velicer 2009; Rendueles *et al*. 2015b) or co‐aggregate into common fruiting bodies at intercolony borders (Rendueles *et al*. 2015b). Our finding that such kin discriminatory allotypes in many cases coexist already at the millimetre scale suggests that colony‐merger incompatibilities affect fine‐scale patterns of migration and group composition in the soil even more than previously appreciated. Second, another recent study showed that the fitness of *M. xanthus* natural isolates is pervasively frequency dependent in a positive manner, such that any given strain can outcompete most other strains if its local frequency is sufficiently high (Rendueles *et al*. 2015a). One implication of this striking phenomenon is that local dispersal events across distinct social allotype territories should often fail to result in successful invasion due to spatial priority effects that promote the maintenance of allotype diversity across patchily distributed populations.

### Process: possible evolutionary causes and consequences of high colony‐allotype density

The high‐density mosaic of allotype patches revealed by our data does not inherently explain why the traits that cause such incompatibilities rose to high frequency in the first place. Nonetheless, we suggest that high allotype density does have implications for the relative plausibility of hypotheses regarding the evolutionary causes and consequences of colony‐merger incompatibilities in comparison to alternative scenarios such as spatially random allotype distributions or a pattern in which allotype patches were substantially larger than is actually the case.

Regarding evolutionary causation, traits that generate colony‐merger incompatibilities may have become common due to selection specifically because they reduce or prevent colony merger, or for other reasons. Both theoretical considerations and recent empirical findings indicate that kin discrimination phenotypes can readily evolve indirectly as by‐products of differential adaptation of a phenotype other than colony nonmerger *per se* (Grosberg 1988; Rendueles *et al*. 2015b; Stefanic *et al*. 2015; Velicer & Plucain 2016). Thus, it has been proposed that indirect evolution should be the default null hypothesis to explain the origin of traits that reduce colony merger between allotypes in the absence of clear evidence of selection specifically for merger prevention (Rendueles *et al*. 2015b). The large number of swarming allotypes reported here and the ease with which many distinct allotypes can evolve in experimental populations (Rendueles *et al*. 2015b) both suggest that a large number of genetic loci can generate colony incompatibilities. In turn, a large number of incompatibility loci increase the probability of new swarming allotypes evolving as pleiotropic by‐products of selection on traits other than territorial kin discrimination *per se*.

Yet, despite the plausibility of indirect‐causation hypotheses, it has also been noted that in some colony encounters, merger prevention could be beneficial for colonies of genotypes that would be at a competitive disadvantage to a genetically distinct neighbouring colony only if the colonies were to merge (Vos & Velicer 2009; Rendueles *et al*. 2015a,b). Thus, direct selection for colony‐merger prevention may play some role in generating and maintaining allotype diversity. A high density of spatially structured allotypes should increase the frequency with which this scenario occurs (relative to low allotype density or a random distribution). Further investigations are necessary to assess the relative contributions of indirect evolution vs. direct selection for traits that reduce colony merger in determining the high density and diversity of allotype patches found in *M. xanthus* soil populations.

High allotype patch density is likely to have consequences for *M. xanthus* fitness landscapes by increasing the importance of traits that uniquely or disproportionately affect the fitness outcomes of encounters between colonies of distinct allotypes. For example, some genotypes that would have a fitness advantage over others if colonies were to freely merge may be under selection to overcome colony incompatibilities to allow penetration into the colonies of genotypes that would be inferior competitors in mixed groups. Likewise, strong group cohesion *via* kin discrimination could protect social groups from invaders dispersing from neighbouring allotypes. Additionally, because allotypic colonies should constitute a barrier to local migration *via* motility, high allotype patch density may increase selection for traits that promote dispersal by other means (Dahl *et al*. 2011), such as carriage by larger organisms.

The fine spatial scale of allotype patches may also have consequences for the average level of cooperation at any given social trait in a population (e.g. fruiting body development). Socially defective cheaters reduce cooperation as they increase in frequency within the groups in which they appear by mutation or migration (Velicer *et al*. 2000; Vulic & Kolter 2001; Fiegna & Velicer 2003; Popat *et al*. 2012). In some scenarios of mutation and migration, merger incompatibilities between colonies have the potential to limit the migration of cheaters across allotype patches and increase average relatedness at cooperation loci (Rendueles *et al*. 2015a). If the rate at which cheaters would migrate across a population in the absence of kin discriminatory barriers were sufficiently high relative to the rate at which new cheaters arise by mutation, colony‐merger incompatibilities may indirectly promote higher average levels of cooperation in a population than would be maintained in the absence of such incompatibilities. However, understanding the relative contributions of colony‐merger incompatibilities, other forces that limit intergroup migration, mutation rate to cheating phenotypes and group‐level selection against cheaters to determining average levels of cheating in natural soil populations requires further investigation.

### Dispersal and structure across larger spatial scales

There has been a major shift in thinking regarding the potential for dispersal limitation to shape microbial biogeography. The famous Baas‐Becking hypothesis, formulated many decades ago, proposed that only local variation in selective conditions generates structure in natural microbial populations (Beijerinck 1913; Baas‐Becking 1934). However, growing evidence indicates that limited dispersal *per se* strongly contributes to population structure and that the Baas‐Becking hypothesis is incorrect, especially when applied to intraspecific variation (Cho & Tiedje 2000; Fenchel 2003; Whitaker *et al*. 2003; Vos & Velicer 2008a; Keymer *et al*. 2009; Oakley *et al*. 2010; Bahl *et al*. 2011). Moreover, even at distances across which dispersal eventually occurs given sufficient time (Bell 2010), average rates of adaptation to local conditions can exceed the rates at which genetic variation is introduced *via* dispersal across locales (Nekola & White 1999).

Our results reveal both spatial structure and some degree of dispersal across all spatial scales examined. First, although the frequency of genetic identity among randomly selected individuals decreases greatly as a function of distance, individuals with identical sequence types can nonetheless occasionally be found up to metres apart from one another (Table 1). At a larger scale, representation of multiple clades within each of the km‐scale sites indicates that dispersal events have occurred across such distances in the past.

Alongside this evidence for some degree of dispersal, other considerations suggest that the dispersal rate from metre to kilometre scales may be limited. First, a previous study presented evidence of limited *M. xanthus* dispersal beginning at a scale of ~100 km (but did not examine below that scale) (Ramette & Tiedje 2007; Vos & Velicer 2008a), suggesting that dispersal may also be limited at somewhat smaller scales. Second, no sequence type in this study was found at more than one of the three sites separated by ~10 km, yet extremely similar STs were found at different km‐scale sites [e.g. ST14 (GH) and ST15 (MC); ST17 (GH) and ST16/25 (KF); ST09 (KF) and ST12 (GH); ST07 (KF) and ST08 (MC); Table 1, Fig. S1, Supporting information]. Unless the slight divergence between these STs represents very strong local adaptation specific to the respective km‐scale sites (such that cells with the similar ST from another km‐site would be rapidly lost by selection upon dispersal), the fact that one ST can be found among independent isolates at the metre scale and below but not across km‐scale sites suggests that dispersal across the km scale is limited.

Thus, it appears plausible that population structure at m to km scales is generated by a combination of stochastic divergence resulting from limited dispersal and local adaptation to heterogeneous environmental conditions, which has been shown to occur down to the metre scale in microbial systems (Belotte *et al*. 2003; Vos *et al*. 2009; Koskella & Meaden 2013; Kraemer & Kassen 2015). Two patterns in our own data may be suggestive of local adaptation. First, Clade 1 and Clade 2 lineages are both represented within each of the three kilometre‐scale sampling sites but are nonetheless locally clustered within those sites rather than randomly scattered (Fig. 1). Because dispersal at the clade level clearly occurs across the km‐scale sites, clade‐level clustering within those sites may reflect patterns of local adaptation. Another intriguing pattern in our data suggests local adaptation across km‐scale sites, namely the complete absence of Clade 3 members from the GH site. This extremely nonrandom pattern is particularly surprising in the light of the fact that Clade 3 is the oldest clade and its lineages have therefore had the longest opportunity to disperse. Given that lineages of both other younger clades have dispersed across and are common at all three km‐scale sites, the absence of Clade 3 lineages from GH suggests that this clade may be maladapted to selective conditions there.

Whatever the precise relative contributions of dispersal limitation and local adaptation to population structure at any given scale may be, *M. xanthus* cells that actively migrate across local terrain at the millimetre or centimetre scale or are dispersed metres or more by external agents are likely to encounter socially incompatible genotypes.

## Competing interests

The authors declare no conflict of interest.

All authors contributed to the development of concepts presented here. S.A.K. provided field data and samples. S.A.K. conducted genetic screening. S.A.K., S.W. and F.F. conducted analyses. S.A.K., S.W. and G.J.V. wrote the original manuscript. All authors contributed to revisions of the manuscript.

## Data accessibility

Individual genotyping and phenotyping data, as well as scripts and input data for statistical analyses, are provided at the Dryad Digital Repository: doi:10.5061/dryad.cn6v1.

## Supporting information


**Fig. S1** Maximum‐likelihood (ML) tree depicting three major clades in our sample set.
**Fig. S2** Maximum‐likelihood inference among the 147 individual five‐gene concatemer sequences depicted in a circle tree.
**Fig. S3** Splits decomposition analysis supports the basic clade relationships among the 26 unique sequence types inferred by the maximum‐likelihood tree.
**Table S1** Genotypes at five highly variable loci in the *M. xanthus* genomes as well as resulting compound sequence types (STs) for each of the 147 natural isolates sorted by their respective geographic sampling scale.
**Table S2** Tests for selection and recombination for single gene sequence alignments.
