# Supplementary material for: The biogeography of kin discrimination across microbial neighbourhoods
Source: Mol Ecol. 2016 Sep 23;25(19):4875–88. doi: 10.1111/mec.13803 (PMC5054864; doi:10.1111/mec.13803)
Supplement: Supplementary file 1 — Fig. S1 Maximum‐likelihood (ML) tree depicting three major clades in our sample set. Fig. S2 Maximum‐likelihood inference among the 147 individual five‐gene concatemer sequences depicted in a circle tree. Fig. S3 Splits decomposition analysis supports the basic clade relationships among the 26 unique sequence types inferred by the maximum‐likelihood tree. Table S1 Genotypes at five highly variable loci in the M. xanthus genomes as well as resulting compound sequence types (STs) for each of the 147 natural isolates sorted by their respective geographic sampling scale. Table S2 Tests for selection and recombination for single gene sequence alignments. [file MEC-25-4875-s001.docx]

**Appendix S1**

**Susanne A. Kraemer^1^, Sébastien Wielgoss^1^, Francesca Fiegna and Gregory J. Velicer**

^1^ Equal contribution as first author.

**The biogeography of kin discrimination across microbial neighbourhoods**

Supporting figures (Figs. S1, S2 and S3) and tables (Tables S1 and S2)


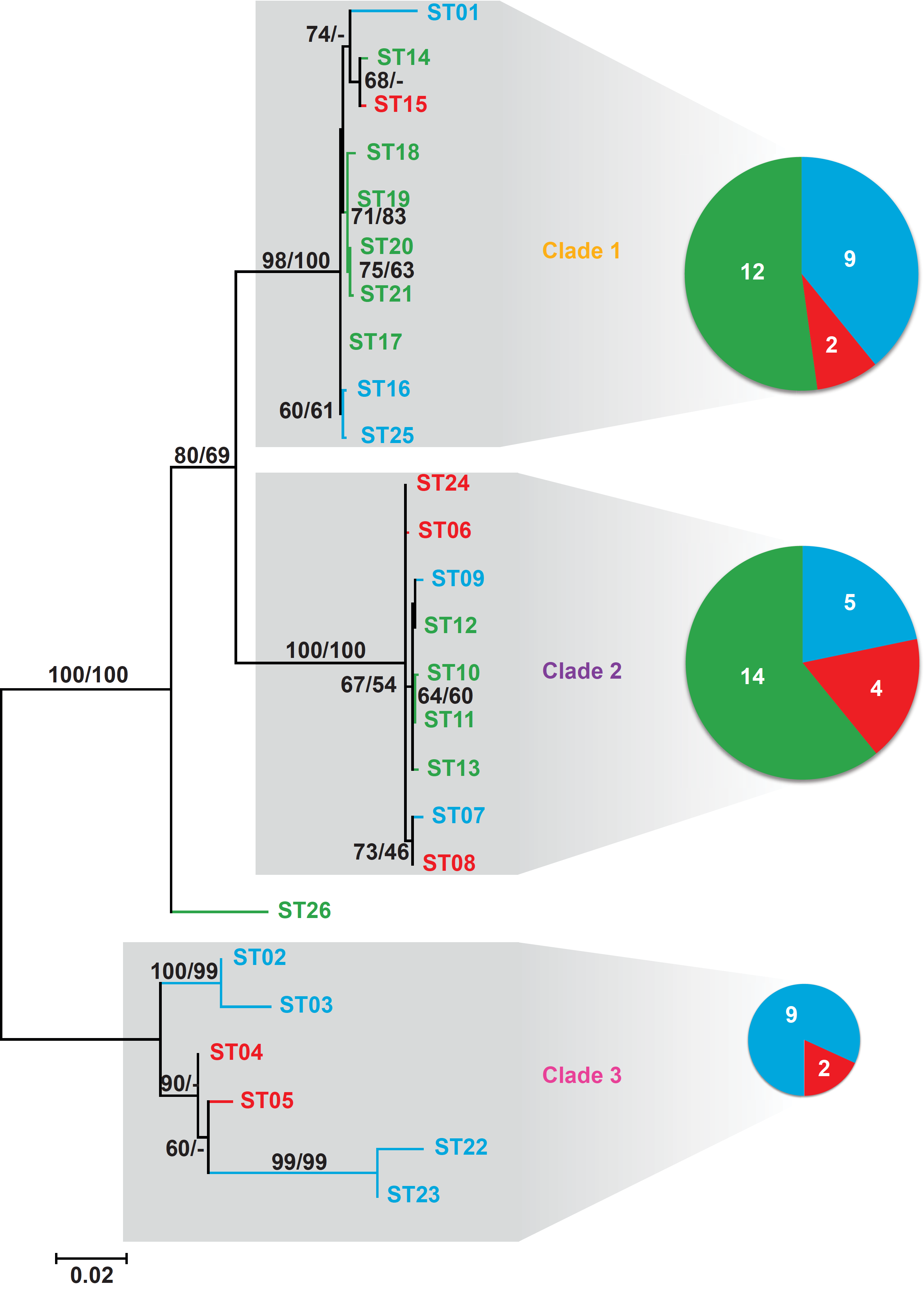


**Figure S1. Maximum likelihood (ML) tree depicting three major clades in our sample set.** Based on ML inference among the 26 unique five-gene sequence types (STs, Table S1, Supporting information) the presence of three clades was revealed. Each ST is unique to one of the three sampling sites at the kilometer scale (Fig. 1; GH – green; KF – blue; and MC – red). Scaled pie charts highlight the numbers of fruiting bodies from each km-scale site in which members of a given clade were found. Bootstrap values on the branches of the tree are based on 1000 pseudo-replicates and represent the statistical support for maximum-likelihood inference (number on the left-hand side) and an alternative ClonalFrame analysis, which also accounts for possible recombination events (number on the right-hand side).


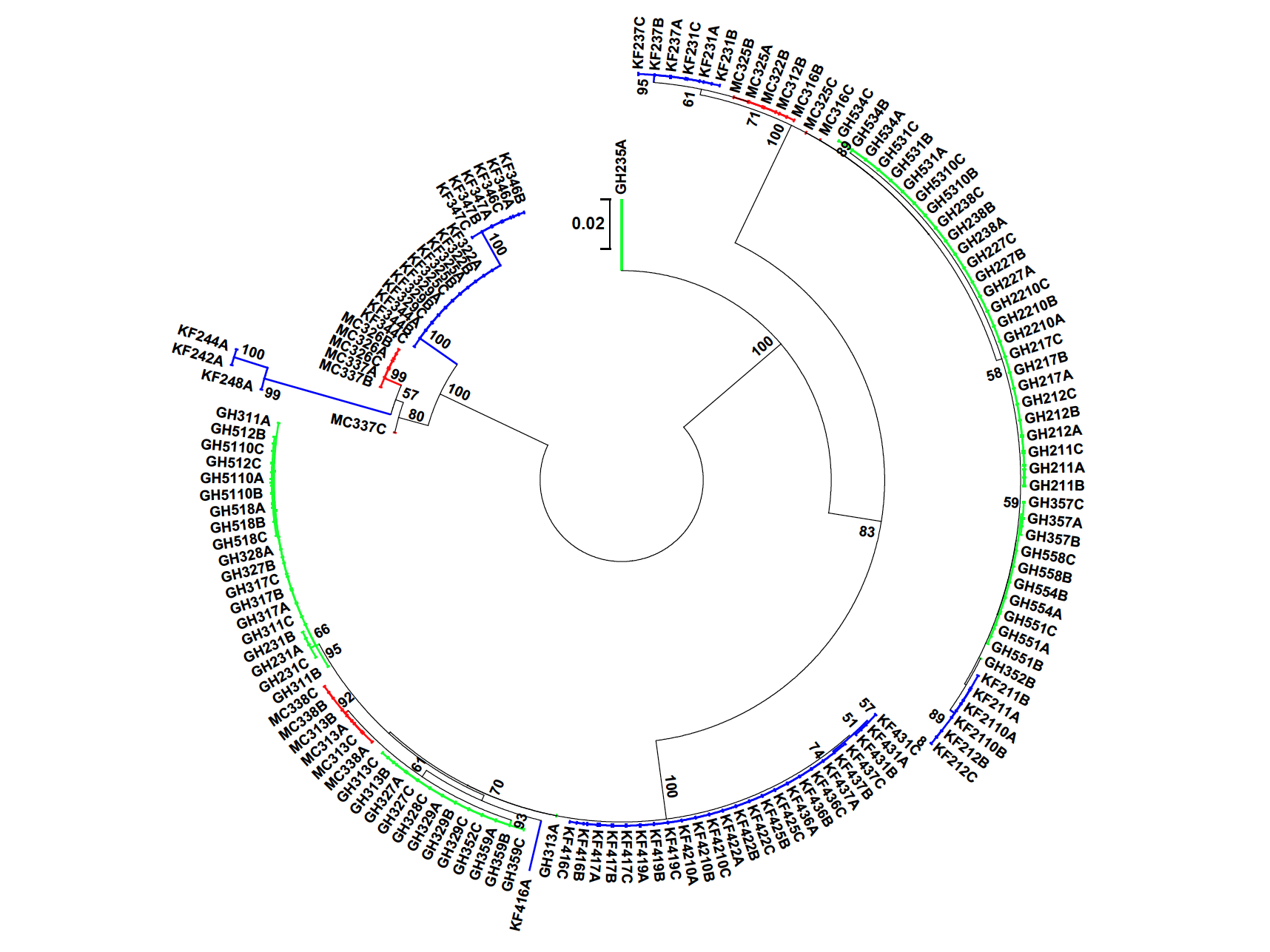


**Figure S2. Maximum likelihood inference among the 147 individual five-gene concatemer sequences depicted in a circle tree.** Analogous to Fig. S1 (Supporting information) each ST is unique to one of the three sampling sites at the kilometer scale (GH – green; KF – blue; and MC – red). Bootstrap values on the branches were derived from 100 bootstrap pseudo-replicates.


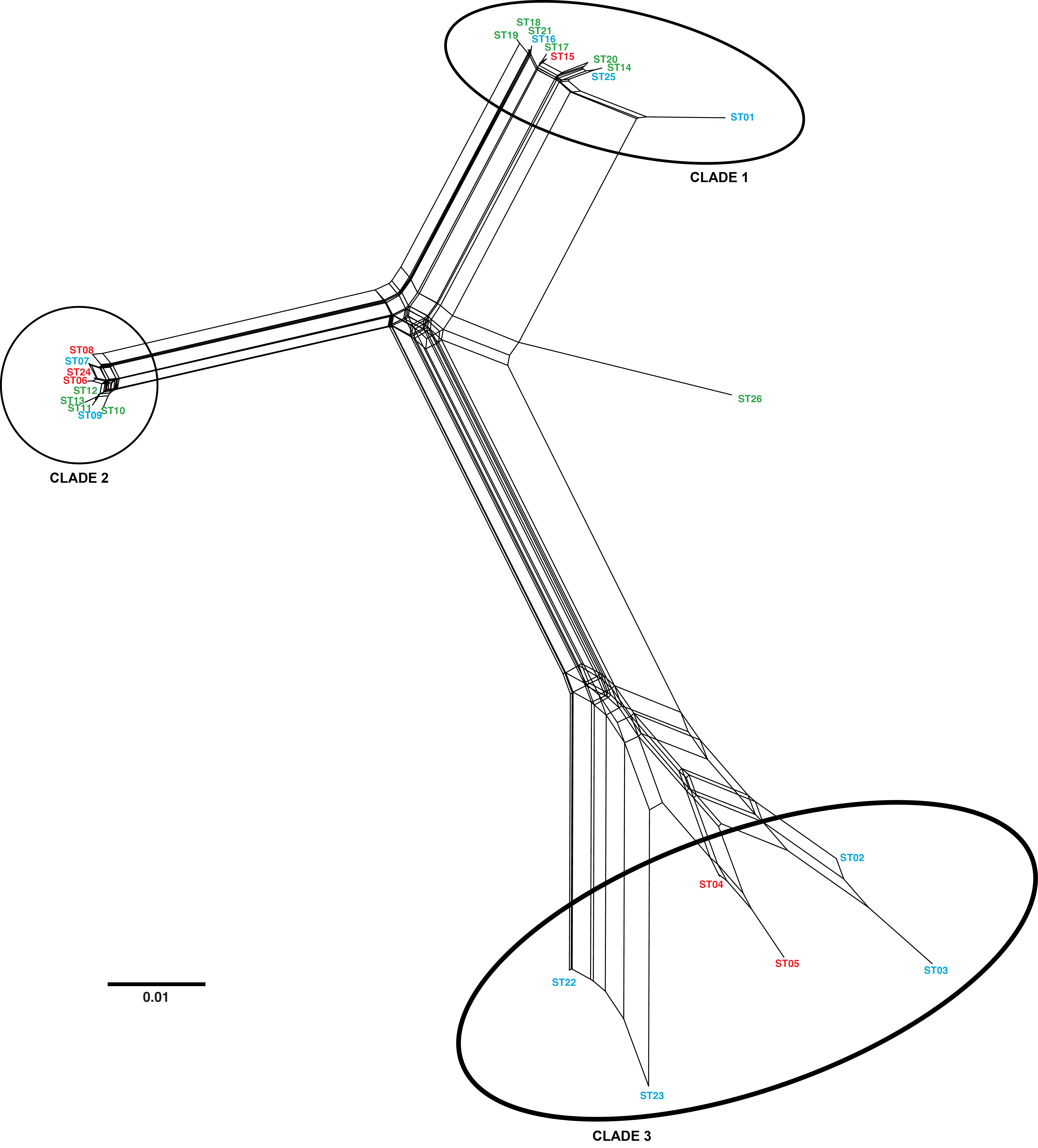


**Figure S3. Splits decomposition analysis supports the basic clade relationships among the 26 unique sequence types inferred by the maximum likelihood tree.** Each of the three sampling sites at the kilometer scale is color-coded (GH – green; KF – blue; MC – red).

**Table S1. Genotypes at five highly variable loci in the *M. xanthus* genomes as well as the resulting concatenated sequence type (STs) for each of 147 natural isolates sorted by their geographic sampling locations.**

| km-scale | m-scale | cm-scale | mm-scale | µm-scale | Sequence type (ST) ^*^ | MXAN 5783^*^  *^pilA^* | MXAN 0128^1^  previously  *0396* | MXAN 1277^*^ | MXAN 0533^*^ | MXAN 4405^*^ |
| --- | --- | --- | --- | --- | --- | --- | --- | --- | --- | --- |
| GH | 2 | 1 | 1 | A | 13 | 5 | 6 | 1 | 2 | 4 |
| GH | 2 | 1 | 1 | B | 13 | 5 | 6 | 1 | 2 | 4 |
| GH | 2 | 1 | 1 | C | 13 | 5 | 6 | 1 | 2 | 4 |
| GH | 2 | 1 | 2 | A | 13 | 5 | 6 | 1 | 2 | 4 |
| GH | 2 | 1 | 2 | B | 13 | 5 | 6 | 1 | 2 | 4 |
| GH | 2 | 1 | 2 | C | 13 | 5 | 6 | 1 | 2 | 4 |
| GH | 2 | 1 | 7 | A | 13 | 5 | 6 | 1 | 2 | 4 |
| GH | 2 | 1 | 7 | B | 13 | 5 | 6 | 1 | 2 | 4 |
| GH | 2 | 1 | 7 | C | 13 | 5 | 6 | 1 | 2 | 4 |
| GH | 2 | 2 | 7 | A | 13 | 5 | 6 | 1 | 2 | 4 |
| GH | 2 | 2 | 7 | B | 13 | 5 | 6 | 1 | 2 | 4 |
| GH | 2 | 2 | 7 | C | 13 | 5 | 6 | 1 | 2 | 4 |
| GH | 2 | 2 | 10 | A | 13 | 5 | 6 | 1 | 2 | 4 |
| GH | 2 | 2 | 10 | B | 13 | 5 | 6 | 1 | 2 | 4 |
| GH | 2 | 2 | 10 | C | 13 | 5 | 6 | 1 | 2 | 4 |
| GH | 2 | 3 | 1 | A | 18 | 6 | 8 | 1 | 1 | 4 |
| GH | 2 | 3 | 1 | B | 18 | 6 | 8 | 1 | 1 | 4 |
| GH | 2 | 3 | 1 | C | 18 | 6 | 8 | 1 | 1 | 4 |
| GH | 2 | 3 | 5 | A | 26 | 8 | 10 | 9 | 2 | 9 |
| GH | 2 | 3 | 8 | A | 13 | 5 | 6 | 1 | 2 | 4 |
| GH | 2 | 3 | 8 | B | 13 | 5 | 6 | 1 | 2 | 4 |
| GH | 2 | 3 | 8 | C | 13 | 5 | 6 | 1 | 2 | 4 |
| GH | 3 | 1 | 1 | A | 19 | 6 | 9 | 1 | 1 | 5 |
| GH | 3 | 1 | 1 | B | 19 | 6 | 9 | 1 | 1 | 5 |
| GH | 3 | 1 | 1 | C | 19 | 6 | 9 | 1 | 1 | 5 |
| GH | 3 | 1 | 3 | A | 17 | 6 | 6 | 1 | 1 | 5 |
| GH | 3 | 1 | 3 | B | 14 | 6 | 2 | 1 | 1 | 5 |
| GH | 3 | 1 | 3 | C | 14 | 6 | 2 | 1 | 1 | 5 |
| GH | 3 | 1 | 7 | A | 19 | 6 | 9 | 1 | 1 | 5 |
| GH | 3 | 1 | 7 | B | 19 | 6 | 9 | 1 | 1 | 5 |
| GH | 3 | 1 | 7 | C | 19 | 6 | 9 | 1 | 1 | 5 |
| GH | 3 | 2 | 7 | A | 14 | 6 | 2 | 1 | 1 | 5 |
| GH | 3 | 2 | 7 | B | 19 | 6 | 9 | 1 | 1 | 5 |
| GH | 3 | 2 | 7 | C | 14 | 6 | 2 | 1 | 1 | 5 |
| GH | 3 | 2 | 8 | A | 19 | 6 | 9 | 1 | 1 | 5 |
| GH | 3 | 2 | 8 | C | 14 | 6 | 2 | 1 | 1 | 5 |
| GH | 3 | 2 | 9 | A | 14 | 6 | 2 | 1 | 1 | 5 |
| GH | 3 | 2 | 9 | B | 14 | 6 | 2 | 1 | 1 | 5 |
| GH | 3 | 2 | 9 | C | 14 | 6 | 2 | 1 | 1 | 5 |
| GH | 3 | 5 | 2 | B | 12 | 5 | 6 | 1 | 1 | 5 |
| GH | 3 | 5 | 2 | C | 14 | 6 | 2 | 1 | 1 | 5 |
| GH | 3 | 5 | 7 | A | 10 | 5 | 4 | 1 | 2 | 7 |
| GH | 3 | 5 | 7 | B | 10 | 5 | 4 | 1 | 2 | 7 |
| GH | 3 | 5 | 7 | C | 10 | 5 | 4 | 1 | 2 | 7 |
| GH | 3 | 5 | 9 | A | 14 | 6 | 2 | 1 | 1 | 5 |
| GH | 3 | 5 | 9 | B | 14 | 6 | 2 | 1 | 1 | 5 |
| GH | 3 | 5 | 9 | C | 14 | 6 | 2 | 1 | 1 | 5 |
| GH | 5 | 1 | 2 | B | 20 | 6 | 9 | 1 | 1 | 7 |
| GH | 5 | 1 | 2 | C | 21 | 6 | 9 | 1 | 2 | 7 |
| GH | 5 | 1 | 8 | A | 20 | 6 | 9 | 1 | 1 | 7 |
| GH | 5 | 1 | 8 | B | 20 | 6 | 9 | 1 | 1 | 7 |
| GH | 5 | 1 | 8 | C | 20 | 6 | 9 | 1 | 1 | 7 |
| GH | 5 | 1 | 10 | A | 20 | 6 | 9 | 1 | 1 | 7 |
| GH | 5 | 1 | 10 | B | 20 | 6 | 9 | 1 | 1 | 7 |
| GH | 5 | 1 | 10 | C | 20 | 6 | 9 | 1 | 1 | 7 |
| GH | 5 | 3 | 1 | A | 13 | 5 | 6 | 1 | 2 | 4 |
| GH | 5 | 3 | 1 | B | 13 | 5 | 6 | 1 | 2 | 4 |
| GH | 5 | 3 | 1 | C | 13 | 5 | 6 | 1 | 2 | 4 |
| GH | 5 | 3 | 4 | A | 13 | 5 | 6 | 1 | 2 | 4 |
| GH | 5 | 3 | 4 | B | 13 | 5 | 6 | 1 | 2 | 4 |
| GH | 5 | 3 | 4 | C | 13 | 5 | 6 | 1 | 2 | 4 |
| GH | 5 | 3 | 10 | B | 13 | 5 | 6 | 1 | 2 | 4 |
| GH | 5 | 3 | 10 | C | 13 | 5 | 6 | 1 | 2 | 4 |
| GH | 5 | 5 | 1 | A | 11 | 5 | 6 | 1 | 2 | 7 |
| GH | 5 | 5 | 1 | B | 11 | 5 | 6 | 1 | 2 | 7 |
| GH | 5 | 5 | 1 | C | 11 | 5 | 6 | 1 | 2 | 7 |
| GH | 5 | 5 | 4 | A | 11 | 5 | 6 | 1 | 2 | 7 |
| GH | 5 | 5 | 4 | B | 11 | 5 | 6 | 1 | 2 | 7 |
| GH | 5 | 5 | 8 | B | 11 | 5 | 6 | 1 | 2 | 7 |
| GH | 5 | 5 | 8 | C | 11 | 5 | 6 | 1 | 2 | 7 |
| KF | 2 | 1 | 1 | A | 9 | 4 | 6 | 1 | 1 | 2 |
| KF | 2 | 1 | 1 | B | 9 | 4 | 6 | 1 | 1 | 2 |
| KF | 2 | 1 | 2 | B | 9 | 4 | 6 | 1 | 1 | 2 |
| KF | 2 | 1 | 2 | C | 9 | 4 | 6 | 1 | 1 | 2 |
| KF | 2 | 1 | 10 | A | 9 | 4 | 6 | 1 | 1 | 2 |
| KF | 2 | 1 | 10 | B | 9 | 4 | 6 | 1 | 1 | 2 |
| KF | 2 | 3 | 1 | A | 7 | 3 | 9 | 1 | 1 | 4 |
| KF | 2 | 3 | 1 | B | 7 | 3 | 9 | 1 | 1 | 4 |
| KF | 2 | 3 | 1 | C | 7 | 3 | 9 | 1 | 1 | 4 |
| KF | 2 | 3 | 7 | A | 7 | 3 | 9 | 1 | 1 | 4 |
| KF | 2 | 3 | 7 | B | 7 | 3 | 9 | 1 | 1 | 4 |
| KF | 2 | 3 | 7 | C | 7 | 3 | 9 | 1 | 1 | 4 |
| KF | 2 | 4 | 2 | A | 22 | 7 | 6 | 6 | 4 | 1 |
| KF | 2 | 4 | 4 | A | 22 | 7 | 6 | 6 | 4 | 1 |
| KF | 2 | 4 | 8 | A | 23 | 7 | 7 | 6 | 4 | 1 |
| KF | 3 | 2 | 2 | A | 2 | 2 | 5 | 5 | 1 | 3 |
| KF | 3 | 2 | 2 | B | 2 | 2 | 5 | 5 | 1 | 3 |
| KF | 3 | 2 | 5 | A | 2 | 2 | 5 | 5 | 1 | 3 |
| KF | 3 | 2 | 5 | B | 2 | 2 | 5 | 5 | 1 | 3 |
| KF | 3 | 2 | 5 | C | 2 | 2 | 5 | 5 | 1 | 3 |
| KF | 3 | 2 | 9 | A | 2 | 2 | 5 | 5 | 1 | 3 |
| KF | 3 | 2 | 9 | B | 2 | 2 | 5 | 5 | 1 | 3 |
| KF | 3 | 2 | 9 | C | 2 | 2 | 5 | 5 | 1 | 3 |
| KF | 3 | 4 | 4 | A | 2 | 2 | 5 | 5 | 1 | 3 |
| KF | 3 | 4 | 4 | B | 2 | 2 | 5 | 5 | 1 | 3 |
| KF | 3 | 4 | 4 | C | 2 | 2 | 5 | 5 | 1 | 3 |
| KF | 3 | 4 | 6 | A | 3 | 2 | 5 | 5 | 5 | 3 |
| KF | 3 | 4 | 6 | B | 3 | 2 | 5 | 5 | 5 | 3 |
| KF | 3 | 4 | 6 | C | 3 | 2 | 5 | 5 | 5 | 3 |
| KF | 3 | 4 | 7 | A | 3 | 2 | 5 | 5 | 5 | 3 |
| KF | 3 | 4 | 7 | B | 3 | 2 | 5 | 5 | 5 | 3 |
| KF | 3 | 4 | 7 | C | 3 | 2 | 5 | 5 | 5 | 3 |
| KF | 4 | 1 | 6 | A | 1 | 6 | 5 | 1 | 1 | 6 |
| KF | 4 | 1 | 6 | B | 16 | 6 | 4 | 1 | 1 | 6 |
| KF | 4 | 1 | 6 | C | 16 | 6 | 4 | 1 | 1 | 6 |
| KF | 4 | 1 | 7 | A | 16 | 6 | 4 | 1 | 1 | 6 |
| KF | 4 | 1 | 7 | B | 16 | 6 | 4 | 1 | 1 | 6 |
| KF | 4 | 1 | 7 | C | 16 | 6 | 4 | 1 | 1 | 6 |
| KF | 4 | 1 | 9 | A | 16 | 6 | 4 | 1 | 1 | 6 |
| KF | 4 | 1 | 9 | B | 16 | 6 | 4 | 1 | 1 | 6 |
| KF | 4 | 1 | 9 | C | 16 | 6 | 4 | 1 | 1 | 6 |
| KF | 4 | 2 | 2 | A | 16 | 6 | 4 | 1 | 1 | 6 |
| KF | 4 | 2 | 2 | B | 16 | 6 | 4 | 1 | 1 | 6 |
| KF | 4 | 2 | 2 | C | 16 | 6 | 4 | 1 | 1 | 6 |
| KF | 4 | 2 | 5 | B | 16 | 6 | 4 | 1 | 1 | 6 |
| KF | 4 | 2 | 5 | C | 16 | 6 | 4 | 1 | 1 | 6 |
| KF | 4 | 2 | 10 | A | 16 | 6 | 4 | 1 | 1 | 6 |
| KF | 4 | 2 | 10 | B | 16 | 6 | 4 | 1 | 1 | 6 |
| KF | 4 | 2 | 10 | C | 16 | 6 | 4 | 1 | 1 | 6 |
| KF | 4 | 3 | 1 | A | 25 | 6 | 4 | 1 | 1 | 7 |
| KF | 4 | 3 | 1 | B | 25 | 6 | 4 | 1 | 1 | 7 |
| KF | 4 | 3 | 1 | C | 25 | 6 | 4 | 1 | 1 | 7 |
| KF | 4 | 3 | 6 | A | 16 | 6 | 4 | 1 | 1 | 6 |
| KF | 4 | 3 | 6 | B | 16 | 6 | 4 | 1 | 1 | 6 |
| KF | 4 | 3 | 6 | C | 16 | 6 | 4 | 1 | 1 | 6 |
| KF | 4 | 3 | 7 | A | 16 | 6 | 4 | 1 | 1 | 6 |
| KF | 4 | 3 | 7 | B | 16 | 6 | 4 | 1 | 1 | 6 |
| KF | 4 | 3 | 7 | C | 16 | 6 | 4 | 1 | 1 | 6 |
| MC | 3 | 1 | 2 | B | 8 | 3 | 9 | 1 | 2 | 6 |
| MC | 3 | 1 | 3 | A | 15 | 6 | 3 | 1 | 1 | 7 |
| MC | 3 | 1 | 3 | B | 15 | 6 | 3 | 1 | 1 | 7 |
| MC | 3 | 1 | 3 | C | 15 | 6 | 3 | 1 | 1 | 7 |
| MC | 3 | 1 | 6 | B | 8 | 3 | 9 | 1 | 2 | 6 |
| MC | 3 | 1 | 6 | C | 6 | 3 | 4 | 1 | 2 | 6 |
| MC | 3 | 2 | 2 | B | 8 | 3 | 9 | 1 | 2 | 6 |
| MC | 3 | 2 | 5 | A | 8 | 3 | 9 | 1 | 2 | 6 |
| MC | 3 | 2 | 5 | B | 8 | 3 | 9 | 1 | 2 | 6 |
| MC | 3 | 2 | 5 | C | 24 | 3 | 6 | 1 | 2 | 6 |
| MC | 3 | 2 | 6 | A | 5 | 2 | 7 | 7 | 3 | 8 |
| MC | 3 | 2 | 6 | B | 5 | 2 | 7 | 7 | 3 | 8 |
| MC | 3 | 2 | 6 | C | 5 | 2 | 7 | 7 | 3 | 8 |
| MC | 3 | 3 | 7 | A | 5 | 2 | 7 | 7 | 3 | 8 |
| MC | 3 | 3 | 7 | B | 5 | 2 | 7 | 7 | 3 | 8 |
| MC | 3 | 3 | 7 | C | 4 | 2 | 7 | 7 | 1 | 8 |
| MC | 3 | 3 | 8 | A | 15 | 6 | 3 | 1 | 1 | 7 |
| MC | 3 | 3 | 8 | B | 15 | 6 | 3 | 1 | 1 | 7 |
| MC | 3 | 3 | 8 | C | 15 | 6 | 3 | 1 | 1 | 7 |

^*^ Identical coloration highlights identical alleles shared by different clones for either single genes or the compound sequence type (ST).

**Table S2. Tests for selection and recombination for single gene sequence alignments**

| Gene | Model | Alignment length^*^  [bp] | Sequence types | Test for Selection  (p-value)† | Recombination breakpoints‡ |
| --- | --- | --- | --- | --- | --- |
| Mxan_0128 | HKY | 351(354) | 9 | 1.0 | 108**, 227** |
| Mxan_1277 | F81 | 147(149^rc^) | 5 | 0.24 | 76 |
| Mxan_0533 | GTR | 339 | 5 | 1.0 | 76, 216 |
| Mxan_4405 | HKY | 333(335^rc^) | 9 | 1.0 | - |
| Mxan_5783 | GTR | 267(268) | 7 | 1.0 | 103, 156 |

^*^Tests performed on sequences put in frame relative to the reference strain *Myxococcus xanthus* DK1622 and trimmed if needed. Numbers in brackets depict length and orientation of the original sequence alignment. Superscript ^rc^ indicates that the original sequence is oriented as reverse complement. † Test for selection using PARRIS as supported by the Akaike information criterion. ‡Inference of the most likely positions at which a recombination breakpoint is detected in each sequence using GARD as supported by the Akaike information criterion. Asterisks depict significance level of the Kishino-Hasegawa (KH) Test for congruence of phylogenies left and right of the respective recombination breakpoints inferred: **, *p* < 0.01.
